# Divergent northern and southern populations and demographic history of the pearl oyster in the western Pacific revealed with genomic SNPs

**DOI:** 10.1111/eva.12905

**Published:** 2020-01-08

**Authors:** Takeshi Takeuchi, Tetsuji Masaoka, Hideo Aoki, Ryo Koyanagi, Manabu Fujie, Noriyuki Satoh

**Affiliations:** ^1^ Marine Genomics Unit Okinawa Institute of Science and Technology Graduate University Onna Japan; ^2^ Aquaculture Technology Division National Research Institute of Aquaculture, Fisheries Research and Education Agency Tamaki‐cho Japan; ^3^ Mie Prefecture Fisheries Research Institute Shima Japan; ^4^ DNA Sequencing Section Okinawa Institute of Science and Technology Graduate University Onna Japan

**Keywords:** adaptation, genetic resource, genotyping by sequencing, pearl oyster, *Pinctada fucata*, population genomics

## Abstract

In the open ocean without terrain boundaries, marine invertebrates with pelagic larvae can migrate long distances using ocean currents, suggesting reduced genetic diversification. Contrary to this assumption, however, genetic differentiation is often observed in marine invertebrates. In the present study, we sought to explain how population structure is established in the western Pacific Ocean, where the strong Kuroshio Current maintains high levels of gene flow from south to north, presumably promoting genetic homogeneity. We determined the population structure of the pearl oyster, *Pinctada fucata*, in the Indo‐Pacific Ocean using genome‐wide genotyping data from multiple sampling localities. Cluster analysis showed that the western Pacific population is distinct from that of the Indian Ocean, and that it is divided into northern (Japanese mainland) and southern (Nansei Islands, China, and Cambodia) populations. Genetic differentiation of *P. fucata* can be explained by geographic barriers in the Indian Ocean and a local lagoon, and by environmental gradients of sea surface temperature (SST) and oxygen concentration in the western Pacific. A genome scan showed evidence of adaptive evolution in genomic loci, possibly associated with changes in environmental factors, including SST and oxygen concentration. Furthermore, Bayesian simulation demonstrated that the past population expansion and division are congruent with ocean warming after the last glacial period. It is highly likely that the environmental gradient forms a genetic barrier that diversifies *P. fucata* populations in the western Pacific. This hypothesis helps to explain genetic differentiation and possible speciation of marine invertebrates.

## 1 INTRODUCTION

A fundamental question regarding ecological and evolutionary processes of marine species is how differentiation and diversification occur in the open ocean. In marine environments, ocean currents advect pelagic organisms from one locality to another, allowing wide distributions. Marine animals with long pelagic larval periods can potentially disperse hundreds or thousands of kilometers (Shanks, 2009; Shanks, Grantham, & Carr, 2003). This long‐range dispersal enhances connectivity of local populations, maintains gene flow, and large effective population size, thereby reducing genetic differentiation among populations (Palumbi, 1994; Ward, Woodwark, & Skibinski, 1994).

Dispersal of marine organisms is, however, confined by geographic factors such as ocean currents and terrain boundaries, and by environmental conditions such as sea surface temperature (SST), oxygen concentration, and salinity. Steady ocean currents restrict the direction of gene flow between localities, resulting in spatial patterns of population structure (Thomas et al., 2015; Xuereb et al., 2018). Marine population can also be dissociated by land exposure. In particular, marine regression that accompanied the last glacial maximum (LGM) around 23,000–18,000 years ago, affected extant population structures (Hewitt, 2000). Furthermore, there is growing evidence that environmental gradients also serve as barriers that prevent genetic flow between local populations (Benestan et al., 2016; Bernatchez et al., 2019; Sandoval‐Castillo, Robinson, Hart, Strain, & Beheregaray, 2018; Sylvester et al., 2018). Larvae delivered by ocean currents can settle only new localities that provide suitable environmental conditions. Therefore, two populations adapted to different environmental conditions are expected to be genetically isolated. Environmental heterogeneity also drives differentiation in genomic loci related to local adaptation (Guo, Li, & Merilä, 2016).

**Figure 1:**
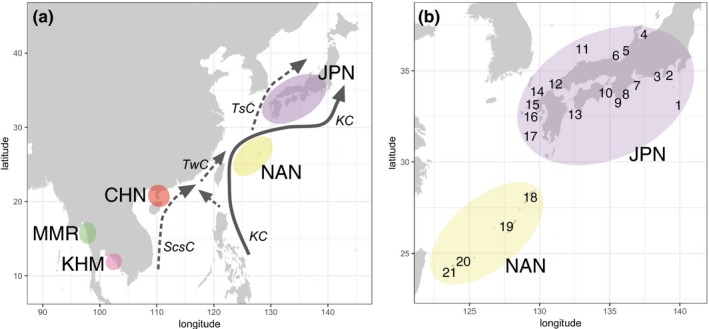
Map of sampling area and ocean currents. (a) Five sampling regions ranging from East to South‐East Asia include Honshu, Shikoku, Kyushu, and marginal islands in Japan (JPN), the Nansei Islands (NAN), China (CHN), Cambodia (KHM), and Myanmar (MMR). A bold arrow indicates a major stream of the Kuroshio Current (KC). Dotted arrows indicate the South China Sea Current (ScsC), the Taiwan Current (TwC), and the Tsushima Current (TsC). (b) Detailed sampling locations in JPN and NAN regions. For more detailed information about sampling locations, see Table 1

In order to understand the impact of environmental factors on differentiation of marine populations, the western Pacific is a fascinating region in which geographic conditions are particularly well studied (Gallagher et al. (2015) for review). The steady Kuroshio Current carries warm water from tropical to subtropical and temperate areas around the Nansei (Ryukyu) and Japanese Archipelagos (Figure 1). Along the Nansei Archipelago, islands are located at less than 100‐km intervals, except for a 200‐km gap between Kume and Miyako Islands. This continuous chain of islands, connected by the Kuroshio Current (mean maximum surface velocity reaches 1.2 m/s (Yang et al., 2015)), allows long‐range distribution of littoral and shallow‐water marine organisms from the Nansei Archipelago to Kyushu, Shikoku, Honshu, and adjacent islands (hereafter we refer to these islands as the "Japanese mainland" for simplicity). In addition, there has been no drastic geological change or ocean current variation since the last glacial period (LGP) in the western Pacific (Gallagher et al., 2015; Voris, 2000). Physical geography, including a continuous chain of islands and ocean currents may have contributed to genetic homogeneity of marine populations in this region.

In fact, some intertidal gastropod species exhibit uniform populations in the western Pacific (Kojima et al., 2006; Yamazaki et al., 2017). It is noteworthy that an intertidal gastropod species *Monodonta confusa*, having only a 3‐day pelagic larval duration (PLD), shows homogeneity from the Nansei Islands to the Japanese mainland (Sasaki, 1985; Yamazaki et al., 2017). This suggests that the Kuroshio Current can transport larvae sufficiently to maintain long‐range population connectivity. In contrast, genetic discontinuity between the Nansei Islands and the Japanese mainland is evident in other animals with various life cycles and PLDs, such as the ostracod *Vargula hilgendorfii* (free‐living adult, but poor swimming ability) (Ogoh & Ohmiya, 2005), the soft shore barnacle *Fistulobalanus albicostatus* (PLD = 5–7 days) (Chang et al., 2017), intertidal gastropods *Mondosa perplexa* and *M. labio* (PLD = 3 days for *M. perplexa*) (Sasaki, 1985; Yamazaki et al., 2017), and a mudskipper *Boleophthalmus pectinirostris* (PLD = 1 month) (Chen, Hong, Chen, Wang, & Zhang, 2015). These observations demonstrate that genetic structure varies among species and reflects more than just pelagic duration. One possible explanation for genetic structure under extensive oceanographic connectivity is that organisms have capabilities specific to environmental conditions; therefore, population structure is restricted by environmental gradients. Despite a number of population genetics studies that have been carried out previously (Chang et al., 2017; Chen et al., 2015; Kojima et al., 2006; Ogoh & Ohmiya, 2005; Sasaki, 1985; Yamazaki et al., 2017), there are no empirical studies that explain population structure of marine organisms in the western Pacific in light of environmental factors, based on genome‐wide variations.

The pearl oyster, *Pinctada fucata*, is a good model to understand genetic differentiation in the western Pacific because of its wide distribution. *P. fucata* is a marine bivalve commonly cultured for pearl production, mainly in Asian countries. Since this species is one of the most valuable aquaculture species, assessment of the genetic structure of natural populations should be highly beneficial for conservation of this resource. *Pinctada* species are typically found in the Indo‐Pacific and western Atlantic tropical and subtropical areas (Cunha, Blanc, Bonhomme, & Arnaud‐Haond, 2011), while *P. fucata* is distributed from low‐latitude tropics to north temperate regions around Japan, representing the northern distributional limit of the genus. *P. fucata* spends 2–3 weeks in its pelagic larval stage during early development (Fujimura, Wada, & Iwaki, 1995). Then it settles on rocky substrates in coastal areas facing the open ocean. Its broadcast spawning strategy, as well as its broad distribution, explains its high‐distance dispersal capability (Yu & Chu, 2006).

To date, the population structure of *P. fucata* in the western Pacific remains controversial. An early biogeographic study demonstrated that the *P. fucata* population in the Nansei area differs from that in the Japanese mainland in frequency of shell color morphs, shell shape, and allozyme variation (Wada, 1984). A DNA marker study using the inter‐simple sequence repeat (ISSR) showed that one PCR fragment was frequently found in individuals from the Nansei Islands, China, Cambodia, and Myanmar, while it was absent from the Japanese mainland (Masaoka & Kobayashi, 2006). Contrarily, no genetic marker was found to distinguish individuals from Japan, China, Vietnam, and Thailand using allozyme loci (Atsumi, Komaru, & Okamoto, 2004). Molecular phylogenetic analyses using complete sequences of 18S and 28S ribosomal RNA and ITS regions did not show significant differentiation among Japanese, Chinese and Cambodian populations (Masaoka & Kobayashi, 2002, 2003, 2005). In addition, artificial fertilization experiments demonstrated that individuals from western Pacific countries produce normal veliger larvae, indicating that they are not isolated reproductively (Atsumi et al., 2004). In summary, population structure may be possible, but genetic analyses with more high‐resolution molecular markers and more comprehensive sampling are necessary to resolve the population structure of *P. fucata* in the Indo‐Pacific Ocean.

In this study, we first conducted restriction site‐associated DNA (RAD) sequencing and retrieved genome‐wide SNPs from *P. fucata* specimens throughout the Indo‐Pacific region to resolve the population structure. Next, in order to identify possible barriers that limit gene flow in the western Pacific, we tested correlations between genetic distance and various environmental factors, including SST, oxygen, salinity, phosphate, nitrate, and carbon dioxide concentrations. Using outlier analysis, genomic loci and genes putatively under non‐neutral evolution were investigated. Finally, we integrated the above results, Bayesian simulations, and admixture analysis to reconstruct the demographic history of *P. fucata* that formed extant populations after the LGP. The results presented here will provide a striking example of genetic differentiation of organisms with high dispersal ability in the open ocean.

## 2 MATERIALS AND METHODS

### 2.1 Sample collection

We collected pearl oysters, *Pinctada fucata*, from five regions in East and South‐East Asia (Figure 1a). Seventeen localities from Honshu, Shikoku, Kyushu, and adjacent islands in Japan (Japanese mainland) are denoted as JPN, and four localities in the Nansei (Ryukyu) Archipelago are labeled as NAN (Figure 1b, Table 1). In order to minimize the effect of artificial admixture in this study, oysters were collected from wild shores or bays, where there were no pearl oyster farms. *P. fucata* specimens from China (CHN), Cambodia (KHM), and Myanmar (MMR) were also analyzed (Figure 1a, Table 1). In total, 178 individuals were collected from 2000 to 2003. An adductor muscle of each individual was sampled and kept in a deep freezer.

**Table 1:** Sample localities

Locality ID	Region	Locality	Latitude	Longitude	Number of samples
JPN_01	Hachijo Island, Japan	Hachijo	33.08	139.76	7
JPN_02	Honshu, Japan	Nishiizu	34.80	138.73	6
JPN_03	Honshu, Japan	Shimizu	34.99	138.60	7
JPN_04	Honshu, Japan	Anamizu	37.17	136.97	7
JPN_05	Honshu, Japan	Tsuruga	35.73	136.06	5
JPN_06	Honshu, Japan	Takahama	35.50	135.54	7
JPN_07	Honshu, Japan	Shima	34.28	136.80	7
JPN_08	Honshu, Japan	Kuki	34.00	136.28	7
JPN_09	Honshu, Japan	Tanabe	33.70	135.34	8
JPN_10	Honshu, Japan	Susami	33.53	135.48	7
JPN_11	Oki Island, Japan	Oki	36.19	133.36	8
JPN_12	Honshu, Japan	Shimonoseki	34.33	130.86	7
JPN_13	Shikoku, Japan	Sukumo	32.89	132.66	8
JPN_14	Tsushima Island, Japan	Tsushima	34.33	129.28	6
JPN_15	Kyushu, Japan	Ohmura	32.88	129.93	8
JPN_16	Kyushu, Japan	Nomozaki	32.58	129.75	7
JPN_17	Kamikoshiki Island, Japan	Kamikoshiki	31.86	129.86	6
NAN_18	Nansei Islands, Japan	Amami	28.18	129.26	8
NAN_19	Nansei Islands, Japan	Zamami	26.20	127.29	4
NAN_20	Nansei Islands, Japan	Ishigaki	24.44	124.12	8
NAN_21	Nansei Islands, Japan	Iriomote	24.33	123.74	8
CHN	Guangdong, China	China	NA	NA	7
KHM	Cambodia	Cambodia	NA	NA	6
MMR	Myanmar	Myanmar	NA	NA	4

### 2.2 DNA extraction, library preparation, and sequencing

Genomic DNA was extracted from tissues using a Maxwell^®^ RSC Blood DNA Kit (Promega) on a Maxwell^®^ RSC Instrument (Promega), according to the manufacturer's standard protocol. Then, it was quantified using a NanoDrop™ 1,000 Spectrophotometer (Thermo Fisher Scientific).

For restriction site‐associated DNA sequence (RAD‐seq) library preparation, genomic DNA was digested with HinfI (NEB) and ligated to a paired‐end sequencing adaptor with a set of 4–8 base barcodes (adapted from Chen et al. (2013)) using T4 ligase (NEB). After removing excess adapters by solid‐phase reversible immobilization (SPRI) purification, libraries were amplified with 15 cycles of PCR using KAPA HiFi HotStart ReadyMix (KAPA). DNA concentration of the library was first estimated with a BioAnalyzer (Agilent), and then measured more precisely with small‐scale sequencing using an Illumina MiSeq Nano kit (Illumina). Libraries were diluted to a normalized concentration before pooling for sequencing, and pooled genotyping by genome reducing and sequencing (GGRS) libraries were sequenced at production scale using Illumina HiSeq 2500 platform (Illumina).

### 2.3 Data processing and genotyping

After sequencing, raw reads with restriction enzyme recognition sites and barcode sequences at both ends were retained with bcl2fastq software (Illumina). Reads were then demultiplexed using reaper and tally, parts of Kraken software tools (Davis, van Dongen, Abreu‐Goodger, Bartonicek, & Enright, 2013), based on custom barcodes to give focused genomic sequence data derived from each individual. Then, adapters and sequences with low quality (average quality below 30 with a 3‐base sliding window) were trimmed with Trimmomatic (version 0.36) (Bolger, Lohse, & Usadel, 2014). Processed reads were mapped to the *P. fucata* reference genome assembly version 2.0 (Takeuchi et al., 2016) using BWA (version 0.7.15) (Li & Durbin, 2009). Individual genotypes were retrieved using BCFtools (version 1.6) mpileup option (Li, 2011). Variant calling was made if a locus had a sequencing coverage depth ≥ 15, and a global minor allele frequency (MAF) rate of at least 10%, reducing the impact of sequencing errors. We removed SNP loci present in fewer than 60% of all individuals. If more than one SNP locus was found within a 150‐bp window (equal to the read length of Illumina sequences), the locus closest to the 5' end of the window was kept for analysis. Then, we retained samples with more than 50% of all loci.

### 2.4 Population structure analyses

We analyzed population structure using model‐free approaches. Principal component analysis (PCA) was performed on all individuals using PLINK 1.9 (Chang et al., 2015). Pairwise genetic distance among localities was estimated with Weir and Cockerham's *F*_ST_ (Weir & Cockerham, 1984) and Nei's genetic distance (Nei, 1972) using StAMPP (Pembleton, Cogan, & Forster, 2013). A neighbor‐joining tree was generated based on Nei's distance matrix in R with the nj function from ape (Paradis, Claude, & Strimmer, 2004). Cluster support of the tree was estimated using the "aboot" function in poppr (Kamvar, Tabima, & Grünwald, 2014) with 1,000 bootstrap replicates.

Population structure of *P. fucata* was further investigated with a model‐based approach using a Bayesian clustering method implemented in fastSTRUCTURE (Raj, Stephens, & Pritchard, 2014). We tested *K* = 2 to *K* = 10 clusters using the simple prior model. Then we chose the number of clusters (*K* = 4) that minimized marginal likelihood using the "chooseK.py" script provided in the fastSTRUCTURE package. We also conducted discriminant analysis of principal components (DAPC) in R using the adegenet library (Jombart, 2008). In order to determine to which cluster each specimen from the western Pacific belonged, samples from MMR were excluded and the number of clusters *K* = 3 was applied to the DAPC. The number of principal components was chosen to optimize the α‐score estimated by "optim.a.score" module in adegenet.

### 2.5 Investigation of outlier loci

We assumed that adaptation to local environments might have occurred in *P. fucata* populations. In order to identify genomic loci exhibiting high genetic differentiation between populations putatively due to selection, we performed three outlier analyses including BayeScan (ver. 2.1) (Foll & Gaggiotti, 2008), PCAdapt (ver. 4.1.0) (Luu, Bazin, & Blum, 2017), and OutFLANK (ver. 0.2) (Whitlock & Lotterhos, 2015). We conducted these conceptually different approaches because cross‐validation using various methods reduces error rates (Villemereuil, Frichot, Bazin, François, & Gaggiotti, 2014). The Bayesian program BayeScan was used to search for loci with extreme *F*_ST_ values that should be explained by selection (Foll & Gaggiotti, 2008). BayeScan was run under default parameters, except for using a prior odds of 200, based on the size of our dataset. Principal component analysis was performed using PCAdapt. Then, a differentiation statistic, Mahalanobis distance, was estimated (Luu et al., 2017). The first principal component (*K* = 1) was used for estimating the test statistics. An R package *q* value (Storey, Bass, Dabney, & Robinson, 2019) was used to calculate *q*‐values in PCAdapt. OutFLANK estimates a likelihood based on a trimmed distribution of F_ST_ to infer the neutral distribution of *F*_ST_ (Whitlock & Lotterhos, 2015). Highest and lowest outlier trimming was set to 0.05 and the minimum heterozygosity required for inclusion was set to 0.1, as the default parameters. For each analysis, loci that satisfied a threshold of the false discovery rate (FDR) or *q*‐value of 0.1 were retained. Finally, loci detected by all three methods were considered outliers.

Genomic regions around outlier loci were surveyed to find coding sequences that might be under selection. We searched gene models located within 1 kb of outlier loci in the *P. fucata* reference genome version 2.0 (Takeuchi et al., 2016). We used a 1‐kb window for physical linkage between SNPs and gene models based on the observation that oysters show a low level of linkage disequilibrium (LD) (Gutierrez et al., 2017). Putative gene models that are subject to selection were searched with BLASTP against the UniProtKB database using an *E*‐value threshold <1e^−5^. UniProt IDs were retrieved from the BLASTP results and used for Gene Ontology annotation (Ashburner et al., 2000) using PANTHER (Mi, Muruganujan, Ebert, Huang, & Thomas, 2019).

### 2.6 Assessment of isolation by environmental factors

We assumed that environmental gradients shape *P. fucata* population structure in the western Pacific. To test this hypothesis, we implemented redundancy analyses (RDAs) using the vegan package in R (Oksanen et al., 2019). Ecological data, including sea surface temperature (SST), oxygen concentration, salinity, phosphate, and nitrate, from 2005 to 2012, were downloaded from the NOAA World Ocean Database (Boyer et al., 2013). Data for carbon dioxide concentration from 2000 to 2018 were obtained from the Surface Ocean CO_2_ Atlas (SOCAT) (Bakker et al., 2016). Maps with the finest longitude/latitude grid resolution (0.25° for SST and salinity, and 1° for oxygen, nitrate, phosphate, nitrate, and CO_2_) were used to retrieve ecological values at 22 sampling localities in the Japanese mainland and the Nansei Islands (JPN_1‐17 and NAN_18‐22). Samples from China, Cambodia, and Myanmar were excluded from the analysis since precise sampling localities were unavailable. Seasonal ecological values (quarterly averages over 8 years) of each environmental factor except CO_2_ were calculated. For CO_2_ concentration, an annual average over 19 years was used. These ecological values were scaled for further analyses. Geographic distances between localities were calculated using the Imap package in R (Wallace, 2012).

Since collinearity between variables can affect RDA (Dormann et al., 2013), correlation among variables, including environmental factors and geographic distance, was tested. First, we assessed correlation between geographic distance and annual mean differences in environmental factors among localities (Figure S2). We selected localities located along the Kuroshio Current (JPN_1, 2, 3, 7, 8, 9, 10, 13, NAN_18, 19, 20, and 21) because direct distances between JPN localities in Pacific coast and in Japan Sea coast do not represent the distances that marine organisms actually migrate. Simple Mantel tests for distance and ecological factors were assessed using the R ecodist package (Goslee & Urban, 2007) and Pearson correlation with 1,000 permutations. Because distance and environmental factors other than CO_2_ are significantly correlated (see Figure S2 for detail), distance was omitted from RDA. Effect of isolation‐by‐distance was tested independently (see Note S1 for detail). Second, correlations between each pair of environmental factors were checked by estimating Pearson's correlation coefficient in R. Results showed that sea surface temperature (SST) and oxygen concentration are strongly correlated throughout the year (Figure S3). It is reasonable to expect a negative correlation between SST and oxygen concentration because oxygen solubility in water increases at lower temperatures. Thus, we prepared two datasets: (a) without oxygen and (b) without SST in order to analyze SST and oxygen independently. In addition, because there were correlations between seasons for SST, oxygen, and salinity, we separated the datasets for each season. Consequently, we analyzed eight environmental datasets. For genotyping data, we prepared two datasets: (a) all SNPs and (b) non‐neutral SNPs identified using outlier analyses in order to assess which environmental factors are related to the SNPs under selection. We conducted analysis of variance (ANOVA) with 1,000 permutations to assess the global significance of the model and to evaluate the influence of each environmental factor. To control FDR in the multiple testing, *q*‐values were calculated using the Benjamini–Hochberg (BH) method (Benjamini & Hochberg, 1995).

Using RDA, we also investigated SNP loci putatively related to local adaptation, based on the method of Forester, Lasky, Wagner, and Urban (2018). We conducted RDAs with the all‐SNP dataset for eight environmental datasets as mentioned above. Two RDA axes were taken into account for SNP search because at most two environmental factors were significantly correlated with genetic variations in each dataset (see the Results section for detail). Loadings were calculated for each SNP locus, and loci that showed loading ± 3*SD* from the mean loading of RDA axes were regarded as associated with local adaptation.

### 2.7 Modeling scenario for establishment of population structure

In the western Pacific, northern and southern populations were identified (see Results). Next, we focused on how the two populations separated after the last glacial period (LGP). One premise is that *P. fucata* originally inhabited the southern region, and then northern population appeared afterward, because (a) the genus *Pinctada* is mainly distributed in tropical and subtropical areas at present (Cunha et al., 2011), showing that *Pinctada* is a warm‐water taxon, (b) fossils of *P. fucata* are absent during the LGP in Japan (Masuda & Noda, 1976) and their shells are found in middens created about 6,000 years ago in the Japanese mainland (Yamamoto, 1992), indicating that the northern population appeared after the LGP, and (c) the northward direction of the Kuroshio Current has been stable since the LGP (Gallagher et al., 2015), making it impossible for pearl oyster larvae to have passively migrated from north to south. In order to understand how the two populations become established, we hypothesized two scenarios: expansion (scenario 1) and migration (scenario 2). In scenario 1, the ancestral population in the southern area expanded to reach the northern area. Then the southern and northern populations become divided, assuming that neither reduced its population size significantly during the separation. In scenario 2, the size of the southern population is consistent with that of the ancestral population, and a small number of individuals incidentally migrated and settled in the northern area. Unlike scenario 1, scenario 2 assumes that the present northern population was derived from a limited number of founders, experiencing a founder effect.

We investigated these hypotheses using approximate Bayesian computation (ABC) analysis (Beaumont, 2010) implemented in DIYABC (Cornuet et al., 2014). ABC is a flexible method for drawing statistical inference about complex evolutionary scenarios based on genetic data. ABC compares summary statistics of observed and simulated data under evolutionary scenarios, and then infers parameters without explicit likelihood calculations (Beaumont, 2010). We generated 1 million simulated datasets representing demographic scenarios. The simulated datasets were compared to empirical data to assess the likelihood of each scenario. Summary statistics were calculated for the proportion of zero values, the mean and variance of nonzero values, and the mean of the complete distribution for each population. In addition, summary statistics for *F*_ST_ and Nei's distances between populations were used. The posterior probability of each scenario was calculated using a direct approach (Cornuet et al., 2008) using the 500 closest simulated datasets.

### 2.8 Inference of population admixture

We inferred introgression of one population into the other using TreeMix version 1.13 (Pickrell & Pritchard, 2012). The maximum‐likelihood tree was constructed using an SNP window size of 100 and the MMR population as an outgroup. The number of tested migration events varied from 1 to 5. The model fit was quantified by the covariance for each migration event. The plotting function in R was used to visualize the results.

## 3 RESULTS

### 3.1 Data processing

GGRS sequencing resulted in an average of 60,116,594 reads (8,712,692,726 bp) per individual. After quality filtering (Table S1), reads were mapped to the *P. fucata* reference genome version 2.0 (Takeuchi et al., 2016) for variant calling. In total, 163 of the 178 individuals examined and 36,203 bi‐allelic loci satisfied the criteria for inclusion in further analyses. The proportion of missing values in the final dataset was 23.42%.

### 3.2 Population structure

**Figure 2:**
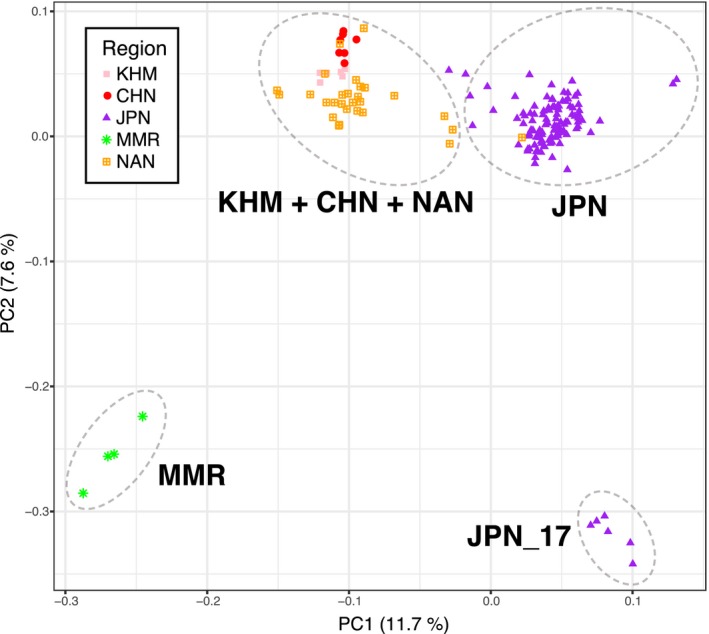
Distinct population structure of *Pinctada fucata* in the Indo‐Pacific area. Principal component analysis (PCA) of all samples. Each dot indicates one individual

Principal component analysis (PCA) of all samples showed a distinct population structure of *P. fucata* in the region (Figures 2 and S1). The first two principal components explained 11.7% and 7.6% of the total variance, respectively. The population at MMR was clearly distinguishable from western Pacific populations in JPN, NAN, CHN, and KHM. The first PC axis separated western Pacific individuals into two major clusters. One is composed of individuals from JPN and another from NAN, CHN, and KHM. Hereafter we denote the JPN population as the “northern population” and the KHM + CHN +NAN population as the “southern population,” according to their geographic distribution. Individuals from JPN_17 (Kamikoshiki Island) were close to those of JPN in terms of PC1 while distinct from the others in PC2.

**Figure 3:**
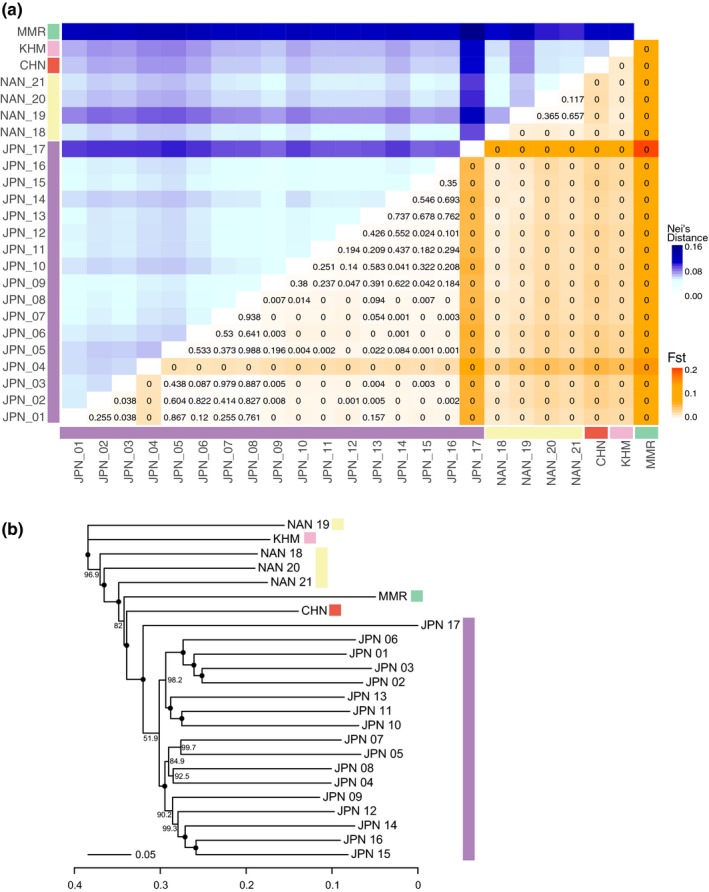
Genetic distances among populations. (a) Pairwise genetic distances (Nei's distance in upper left and F_ST_ in lower right) among sample localities. For all pairwise F_ST_, *q*‐values (FDR adjusted *p*‐values) are show in the tiles. (b) Neighbor‐joining tree based on Nei's distances. Bootstrap values are indicated at the nodes. Black circles indicate 100% bootstrap support

The population structure of *P. fucata* was also confirmed with pairwise values of *F*_ST_ and Nei's distance among populations (Figure 3a, Data S1). Northern populations are close to each other (Nei's distance = 0.04–0.06, *F*_ST_ = 0.00–0.005) except for the populations from Anamizu (JPN_04) and Kamikoshiki Island (JPN_17), which are distinguishable from all other JPN populations (Nei's distance = 0.05–0.05, *F*_ST_ = 0.1–0.02 for JPN_04; Nei's distance = 0.09–0.10, *F*_ST_ = 0.05–0.07 for JPN_17) with significant *q*‐values (*q* = 0.00) for the *F*_ST_. Genetic distances among southern populations (NAN_18–21, CHN, and KHM) showed relatively low values (Nei's distance = 0.04–0.08, *F*_ST_ = 0.00–0.02). The MMR population is distant from other sites (Nei's distance = 0.10–0.16, *F*_ST_ = 0.09–0.19), demonstrating that the population in the Indian Ocean is distinct from that in the western Pacific. A neighbor‐joining tree based on the Nei's distance (Figure 3b) demonstrated that the northern population (JPN) comprises a distinct cluster that is separated from the southern population (NAN, CHN, and KHM). The JPN_17 (Kamikoshiki Island) population was grouped with the northern cluster with a high bootstrap support (100%), although it showed a long branch.

**Figure 4:**
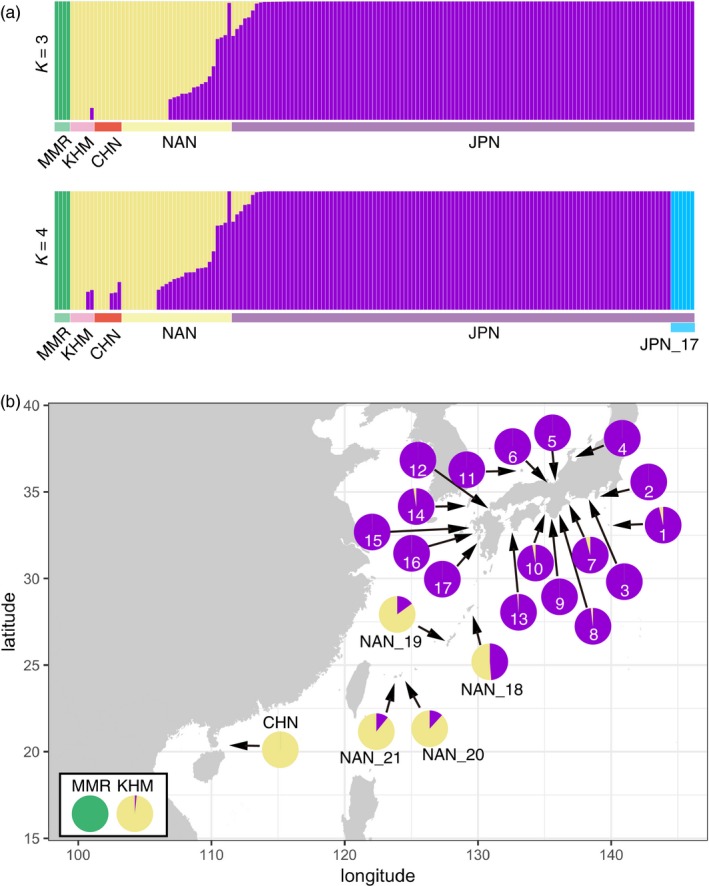
Northern and southern populations of *Pinctada fucata* across the western Pacific are identified using STRUCTURE analysis. (a) Each vertical column represents individual and associated probabilities of assignments to genetic clusters (green, yellow, purple, and cyan). The *Y*‐axis represents the probability that a given individual belongs to the cluster(s) indicated. (b) Geographic configuration of the associated probability of assignments (*K* = 3) for each locality. ID numbers for the JPN population are indicated in circles

In order to explore detailed *P. fucata* population composition, populations were analyzed using fastSTRUCTURE (Raj et al., 2014) (Figure 4). With an optimal value of *K* = 3, each cluster was composed of a population in the Indian Ocean (MMR), the southwestern Pacific (KHM, CHN, and NAN), and the northwestern Pacific (JPN), respectively (Figure 4a, upper panel). The JPN_17 subcluster in the northern population was identified when *K* = 4 (Figure 4a, lower panel). Geographic distributions of the three clusters are shown in Figure 4b. In addition, the result of DAPC confirmed the northern, southern, and Kamikoshiki populations (Figure S1b).

All of these cluster analyses demonstrated consistent population structure: (a) The MMR population is clearly separated from the western Pacific population, (b) in the western Pacific, northern and southern populations are significantly differentiated, and (c) in JPN region, individuals of Kamikoshiki Island (JPN_17) are distinct from the rest of the northern population.

### 3.3 Outlier analyses

**Figure 5:**
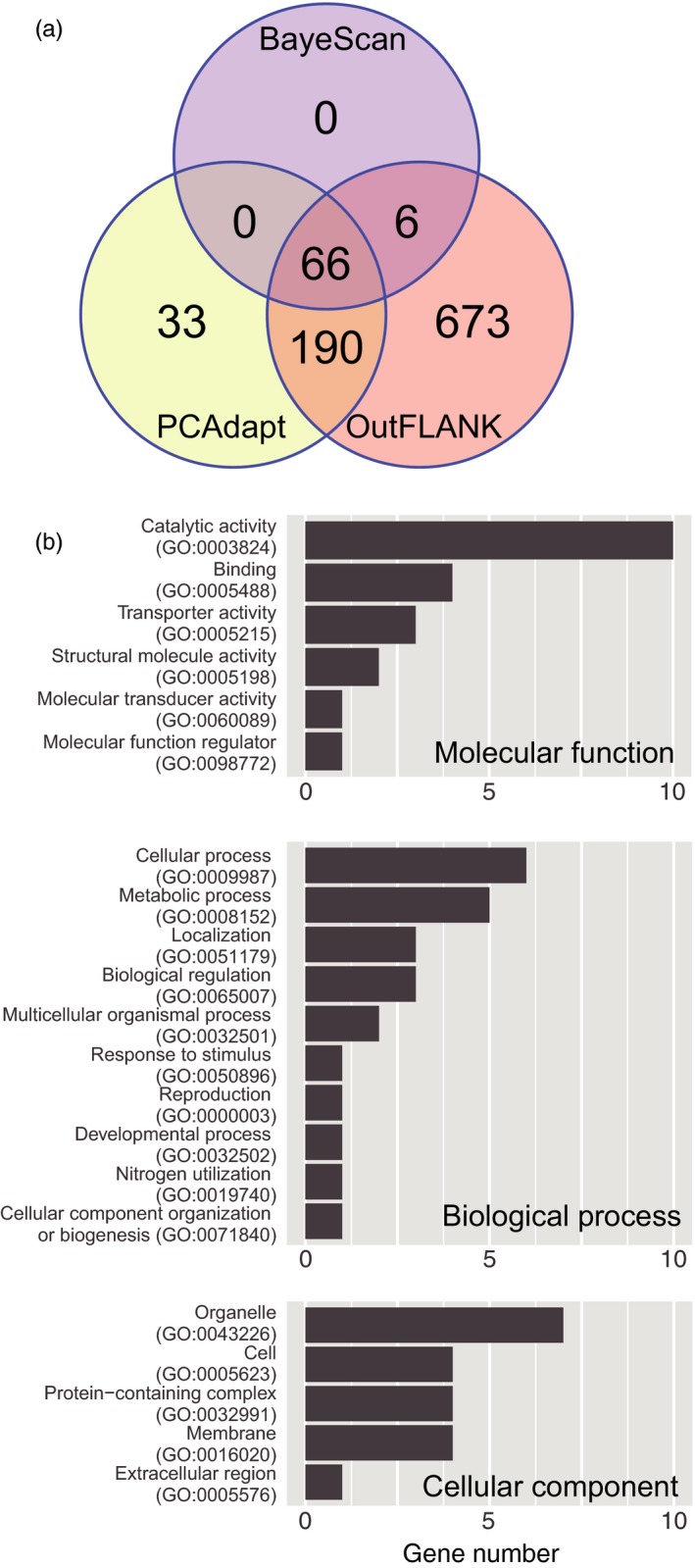
Detection of outlier loci in the *Pinctada fucata* genome. (a) The number of SNP loci identified by each software package. (b) GO functional annotation of gene models that are closely located at outlier SNP loci

The genomic scan demonstrated outlier loci that may be associated with adaptation to local environmental variables such as temperature and oxygen concentration. BayeScan, PCAdapt and OutFLANK analyses detected signatures of non‐neutral selection at 72 (66 + 6), 289 (66 + 190+33), and 935 (6 + 66+190 + 673) loci, respectively (Figure 5a). In order to reduce false‐positive outliers, 66 loci supported by all three methods were regarded as outlier loci.

Assuming that genes physically close to these loci are under non‐neutral selection, we identified 42 gene models located within 1 kb of non‐neutral loci in the reference genome. Among those, 35 gene models were annotated based on results of a BLASTP search against the UniProt database (Table S3). GO annotation showed that 34 genes were classified into six categories of molecular function (Figure 5b). Catalytic activity (10 genes, 47.6%) and binding (4 genes, 19.0%) occupied two‐thirds of the class. The predominant category in biological process is cellular process (6 genes, 25.0%), followed by metabolic process (5 genes, 20.8%), localization (3 genes, 12.6%) and biological regulation (3 genes, 12.5%). In the cellular component class, most genes were categorized as organelle (7 genes, 35.0%).

### 3.4 Isolation by environmental factors

Although northern and southern populations are distinct from each other in the western Pacific, there is no visible terrain boundary separating the populations. We next investigated environmental variables that may disrupt gene flow between the southern and northern populations.

**Figure 6:**
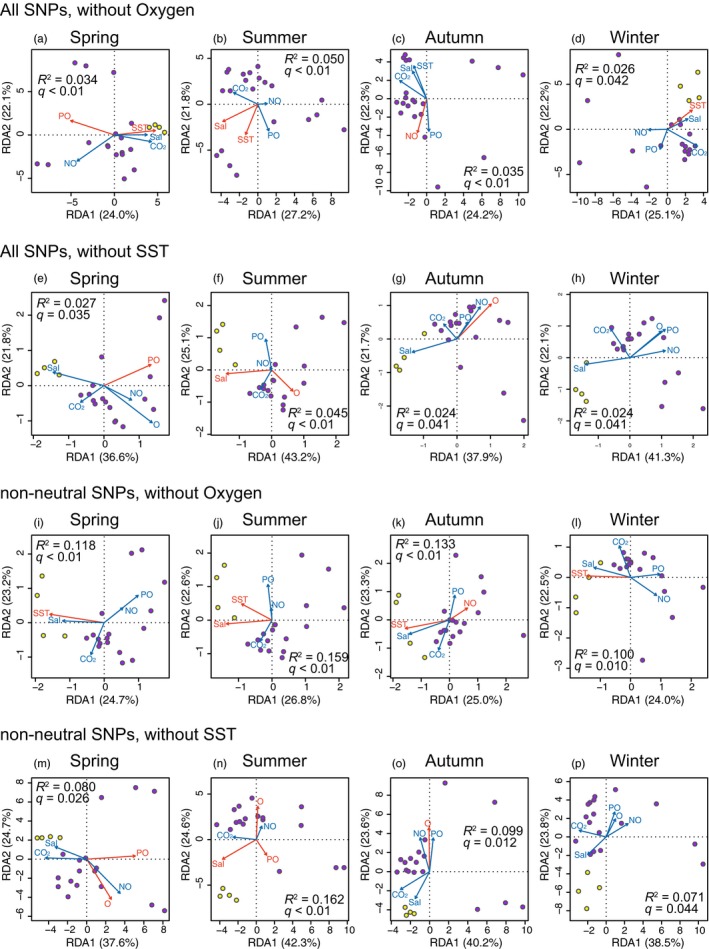
RDA showing significant environmental factors related to genetic differentiation between northern and southern populations. (a–h) All‐SNP dataset. (i–p) Non‐neutral SNP dataset. Arrows represent environmental factors (SST, sea surface temperature; O, oxygen concentration; Sal, salinity; PO, phosphate concentration; NO, nitrate concentration; CO_2_, carbon dioxide concentration). Red arrows indicate significant variables (*q* < 0.05; Table S4). Circles indicate sampling localities of JPN (purple) and NAN (yellow) regions

To test the correlation between genetic variation and environmental variables, a series of RDA was performed (Figure 6, Table S4). When analyzing all SNP datasets (Figure 6a–h), genetic variation was significantly associated with SST in spring, summer, and winter (Figure 6a,b, and d). Other factors including oxygen, salinity, phosphate, were significant in particular seasons. For non‐neutral SNP datasets (Figure 6i–p), SST was the only environmental factor that was significantly related in all four seasons (Figure 6i–l). Oxygen concentration contributed to the differentiation of non‐neutral loci in spring, summer, and autumn datasets (Figure 6m–o). In contrast, contributions of other factors, including salinity, nitrate, phosphate, and CO_2_, were limited. The global RDA was explained more in non‐neutral datasets (*R* ^2^ = 7.1%–16.2%) than in the all‐SNP dataset (*R* ^2^ = 2.4%–5%).

Based on the RDA results, SNP loci possibly associated with adaptation to local environments were investigated (Table S5). In total, 1,049 unique loci were identified and 11 of them were mutually detected by three outlier analyses, BayeScan, PCAdapt, and OutFLANK. We searched genomic regions near the 11 loci (≤1 kb) and found 4 gene models that may be related to adaptation (Table S3).

### 3.5 Population history

**Figure 7:**
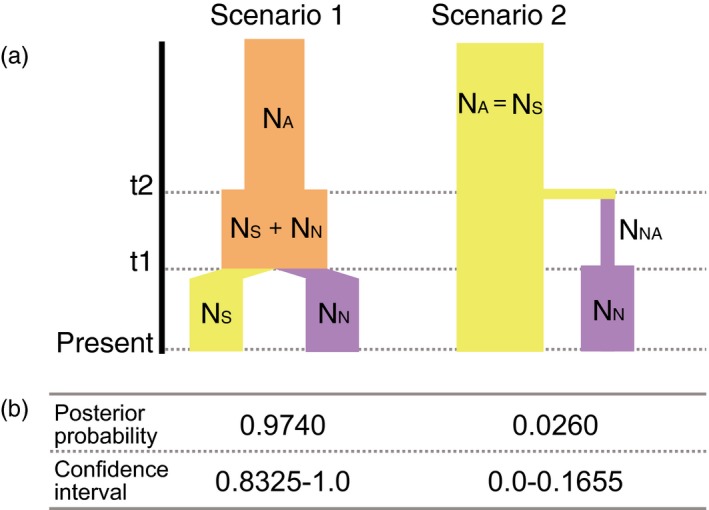
Population history scenarios of *Pinctada fucata* in the western Pacific tested using DIYABC. (a) In scenario 1, the ancestral population (*N*_A_) was increased at *t*_2_, and separated into two populations (*N*_S_ and *N*_N_) without a reduction in the sum of the population size at *t*_1_. Total population size is stable during the subdivision, and no bottleneck event was occurred. In scenario 2, the southern population (*N*_S_) size is stable, while the northern population (*N*_N_) originated from a small number of individuals from the ancestral population (*N*_NA_) derived from *N*_S_ at *t*_2_, and expanded at *t*_1_. A bottleneck in the northern population is assumed in scenario 2. (b) Posterior probability and confidence interval of the scenarios estimated using DIYABC

Next, we addressed the demographic history of *P. fucata* that has shaped current population structure after the LGP in the western Pacific. Using DIYABC, we tested two hypotheses: expansion (scenario 1) and migration (scenario 2) (Figure 7a). Prior scaled parameters, including effective population sizes (*N*) and timing of population events (*t*) were set as shown in Table 2, with uniform distributions. Our results showed that the posterior probability (PP) and the confidence interval (CI) of scenario 1 (PP = 0.974, CI = 0.8325–1.000) are significantly higher than those of scenario 2 (PP = 0.026, CI = 0.000–0.1655; Figure 7b). In other words, no founder effect is evident in establishment of the northern population. This suggests that the ancestral population in the southwest Pacific expanded from the South to the Japanese mainland. Then, gene flow from the South to the North became restricted by environmental changes (discussed below), resulting in two distinct populations.

**Table 2:** Prior parameters for DIYABC simulation

		Prior distribution	Condition
*N*_S_	Size of the southern population at present	[10^3^, 10^7^]	
*N*_N_	Size of the northern population at present	[10^3^, 10^7^]	
*N*_A_	Size of the common ancestral population	[10^3^, 10^7^]	*N*_A_ < *N*_S_
*N*_NA_	Size of the northern ancestral population	[10, 10^4^]	*N*_NA_ < *N*_N_
*t*_1_, *t*_2_	Time point of population events	[10^2^, 10^5^]	*t*_1_ ≦ t_2_

**Figure 8:**
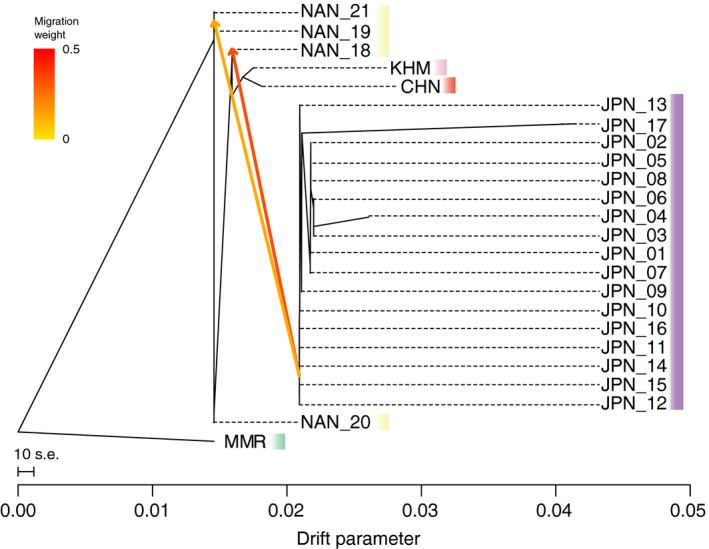
Admixture from the northern population (JPN) to Amami Island is evident using TreeMix. The population tree was built assuming two migration events, using MMR as an outgroup. Arrows indicate inferred admixtures and are colored according to their weights

The TreeMix population graph revealed signs of genetic introgression from the northern population (JPN) to the Nansei Archipelago (NAN) (Figure 8). The proportion of variance in relatedness between populations explained by the model reached 95.9% with two migration events. Evidence of genetic introgression from north to south is congruent with result of fastSTRUCTURE analysis, showing that an average of 48.7% of the genome could be attributed to introgression in Amami (NAN_18) population (Figure 4b). By the present TreeMix analysis, it is not clear from which JPN locality pearl oysters were introduced to NAN population, because genetic differentiation among the JPN population is low.

**Figure 9:**
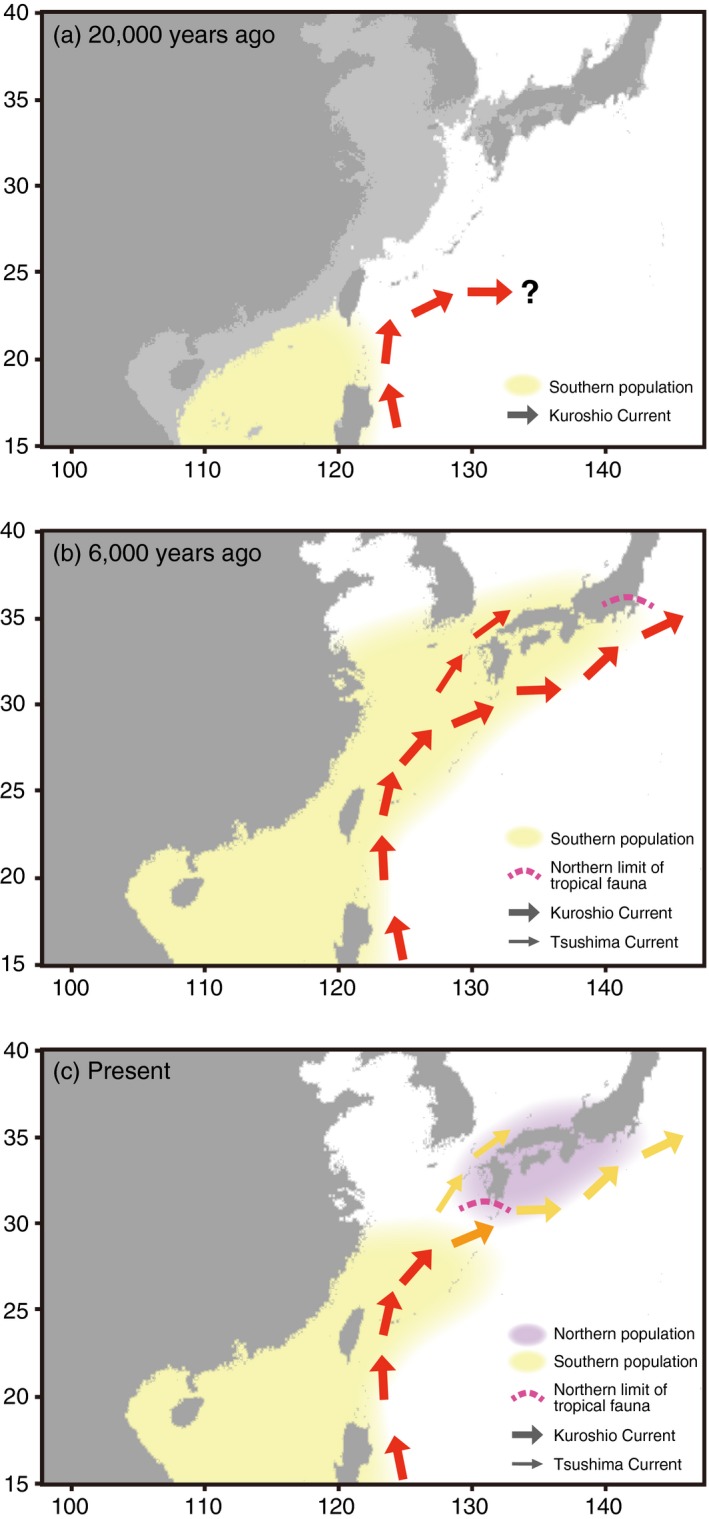
Possible population history of *Pinctada fucata* in the western Pacific. (a) In the last glacial period, the distribution of *P. fucata* was restricted to the South, due to low SST. The light gray area indicates exposed land, assuming that the sea level was 120 m below present. Note that this map is based on modern terrain height and that tectonic change is not considered. (b) The ancestral population expanded to the north, accompanied by increased SST. Tropical species such as hermatypic corals were also distributed to 35°N (dotted line). (c) As the SST gradually decreased to present levels, demographic flow from the South to the North became restricted by this environmental barrier. The northern population was retained, presumably due to adaptation to the local environment

Finally, we reconstructed the possible demographic history of *P. fucata* by considering results of present population genetics (Figure 9). Because they are not able to survive in low‐temperature seawater, *P. fucata* colonized the JPN region after the last glacial period (Masaoka & Kobayashi, 2005) (Figure 9a). Our Bayesian inference results demonstrated that colonization resulted from expansion of the population, rather than incidental migration of small number of individuals. Based on the results, we hypothesized that the ancestral population was expanded (Figure 9b), and then the population became divided into northern and southern populations (Figure 9c).

## 4 DISCUSSION

Population structure of marine invertebrates is determined by biological attributes such as pelagic larval duration and physiology, as well as by seascape features, including geographic setting and environmental variables.

Terrain boundary is one of the apparent barriers that prevents gene flow among marine populations, resulting in vicariance and allopatric differentiation. For example, the Central American Isthmus, which emerged in the late Pliocene (2.8 million years ago), separated the Pacific and the Atlantic Oceans (Coates, McNeill, Aubry, Berggren, & Collins, 2005). Marine organisms diverged into genetically distinct sister clades on both sides of the Isthmus (Lessios, 2008). In the Indo‐Pacific area, a large land mass, corresponding to the Sunda Shelf, was exposed by low sea level during glacial periods of the Pleistocene (Voris, 2000). The appearance of this land mass severed the genetic connection between Indian and Pacific marine populations with long‐lived pelagic larvae, such as starfish and reef fish species (Benzie, 1999; Gaither et al., 2011). The pearl oyster population in MMR may also have been differentiated from the western Pacific population by this terrain boundary (Figures 2, 3, and 4).

The pearl oyster population at Kamikoshiki Island (JPN_17) exemplifies small‐scale, but distinct allopatric differentiation by a geographic barrier. Pearl oysters inhabit a coastal lagoon called Lake Namako‐ike on the shore of the island. The lake is completely isolated from the open sea by a coastal gravel bar with no tidal inlets. The geological barrier, established in mid‐Holocene about 5,000–6,000 years ago (Aramaki, Yamaguchi, & Tanaka, 1976; Woodruff, Donnelly, & Okusu, 2009), genetically isolates the Kamikoshiki population from all others, resulting in unique genetic constitution (Figures 2, 3, and 4a).

Except for the population in Kamikoshiki Island, genetic divergence among localities in the Japanese mainland is low (Figure 3a). One possible explanation is that the pearl oysters have been artificially delivered from one place to another for aquaculture purpose, causing genetic homogenization in the JPN region. However, our sampling localities include islands (JPN_1, 11, and 14) more than 100 km from pearl farming areas (Figure 1b). To the best of our knowledge, there are no records of artificial transfers of pearl oysters in these areas; therefore, local pearl oyster populations of isolated islands cannot have been contaminated by human activities. Thus, the genetic uniformity of population in JPN localities is attributed to natural dispersal of the pearl oyster throughout the region by the Kuroshio and Tsushima Currents (Figure 1a), irrespective of artificial intervention. Genetic uniformity negates the possibility of isolation‐by‐distance to explain the *P. fucata* population in the JPN region (Figure S4). Similarly, the southern population is distributed across a wide area ranging from the South China Sea (KHM and CHN) to the East China Sea (NAN). The wide distribution of the population is maintained by the long‐lived pelagic larvae, together with steady ocean currents. Population uniformity from the Gulf of Thailand to the South China Sea was demonstrated in various marine animals such as the seahorses, *Hippocampus kuda* and *H. trimaculatus* (Lourie, Green, & Vincent, 2005), the acorn barnacle, *Chthamalus malayensis* (Tsang et al., 2012), and the limpet, *Cellana toreuma* (Wang, Ganmanee, Shau‐Hwai, Mujahid, & Dong, 2016). A genetic connection between the South China Sea and Nansei Islands populations is also evident from studies on the toothed top shell, *Monodonta labio* (Yamazaki et al., 2017). These studies support the idea that the South China Sea Current connects KHM and CHN, and that the Kuroshio and Taiwan Currents connect the South China Sea and the NAN area (Figure 1a), maintaining the southern population of *P. fucata*.

### 4.1 Environmental barriers and adaptation to local conditions

As *P. fucata* shows potential to inhabit much of the coastal habitat of the western Pacific, it is speculated that southern and northern populations could also be demographically connected by the Kuroshio Current. In fact, the presence of wild *P. fucata* population in the JPN region confirms that they can migrate from the NAN area to JPN via the Kuroshio Current, at least in the past. In the present ocean, however, the southern and northern populations are clearly differentiated (Figures 2, 3, and 4). In the absence of a geographic barrier, we hypothesize that existing population patterns of *P. fucata* can be explained by their capacity to adapt to local environmental gradients.

As pearl oysters adopt a sessile adult lifestyle, dispersion occurs in the pelagic larval stage from fertilization to settlement, taking 2–3 weeks. Maturation of gonads and spawning of *P. fucata* occurs throughout the year, with a small peak of activity in the winter in the Nansei area (Wada, Komaru, Ichimura, & Kurosaki, 1995). Therefore, the environmental barrier that limits dispersal of larvae from south to north should persist regardless of the season. Among the environmental factors studied here, SST showed the strongest correlation with the genetic distance between the southern and northern populations (Figure 6), a correlation that is evident in all four seasons. The results indicate that the SST gradient is one of the major factors affecting the distribution of *P. fucata* populations in the western Pacific. In fact, seawater temperature directly affects metabolic and physiological activity of pearl oysters (Numaguchi, 1994; Yukihira, Lucas, & Klumpp, 2000). It is noteworthy that the optimum seawater temperature for *P. fucata* in China is significantly higher than that for individuals in Japan (Numaguchi & Tanaka, 1986; Wang, Zhu, Wang, Luo, & Liu, 2012). Furthermore, their growth is considerably affected by water temperature, especially in early life stages such as fertilization, development, and settlement (Numaguchi & Tanaka, 1986; O’Connor & Lawler, 2004; Wang et al., 2012). Larvae originating from the Nansei area may be transported to north, but cannot survive and settle in the lower temperatures of the JPN area.

The northern and southern populations are considered to be adapted to environmental conditions differing in temperature and oxygen level. By comparing genome‐wide SNPs of the two populations, our genome scan analyses, including BayeScan, PCAdapt, and OutFLANK, identified 66 outlier loci, which are the landmark of candidate genes for adaptation. In total, 42 protein‐coding gene models were retrieved from the *P. fucata* reference genome and 35 genes were functionally annotated, based on sequence similarity to known genes in the public database.

Thermal tolerance is associated with physiological oxygen supply and demand. Oxygen deficiency is caused by increasing oxygen demand at high temperatures, and by decreased ventilation and circulation at low temperatures (Pörtner, 2001, 2002). Therefore, ectothermic marine organisms may modify functional properties and density of mitochondria to maximize ATP production efficiency at ambient temperature. In our outlier study, several genes with metabolic functions are identified. For instance, a gene encoding a subunit of succinate dehydrogenase (pfu_aug2.0_10.1_00001.t1), located in the mitochondrial matrix, participates in the electron transport chain, as well as the TCA cycle (Hederstedt & Rutberg, 1981; Yankovskaya et al., 2003). Inverted formin‐2 or INF2 homolog (pfu_aug2.0_1964.1_15330.t1) is involved in mitochondrial fission (Korobova, Ramabhadran, & Higgs, 2013). Notably, this gene is detected not only by all 3 outlier approaches, but also by a genotype–environment association approach using RDA (Table S3). It is possible that gene expression level or activity of these proteins is optimized in response to environmental conditions.

Along with lower seawater temperatures, changes in mitochondrial properties and higher concentrations of oxygen in the environment may cause increasing oxidative stress by cellular reactive oxygen species (ROS) (Abele & Puntarulo, 2004; Guderley, 2004; Viarengo, Canesi, Garcia Martinez, Peters, & Livingstone†, 1995). ROS strongly oxidize all cell components including DNA, proteins, and lipids. The enzymatic defense system against cytotoxic effects of ROS includes superoxide dismutase, catalase, and glutathione peroxidase (Manduzio, Rocher, Durand, Galap, & Leboulenger, 2005). In the present study, however, we did not find any of these genes among adaptation‐related candidates. On the other hand, there is a gene involved in DNA repair, single‐strand selective monofunctional uracil‐DNA glycosylase (pfu_aug2.0_1138.1_28083.t1).

In summary, the present outlier analysis detected a number of genes that may be related to local adaptation. It should be noted that the gene candidate list includes gene models of unknown relevance and that some outliers could be false positives. Meanwhile, information about outliers in the genome suggests potential candidates, the biological function of which should be analyzed in future studies. Experimental approaches such as RNA‐seq to detect differentially expressed genes in different conditions, gene knockdown, or genome editing will be essential to identify genes related to adaptation.

### 4.2 Reconstruction of population history

Extant populations are derived from ancestral populations that experienced environmental changes in the Pleistocene (Hewitt, 2000). Therefore, in order to understand how contemporary population structure was established, it is necessary to consider historical seascape features.

Based on the present population genetics study discussed above, we reconstructed the demographic history of *P. fucata* (Figure 9). During the last glacial period, the SST around the Japanese mainland was 5–6°C lower than at present. The Kuroshio Current was thought to have turned westward at a lower latitude than today (Ujiié, Tanaka, & Ono, 1991; Ujiié, Ujiié, Taira, Nakamura, & Oguri, 2003), but see Lee et al. (2013) and Gallagher et al. (2015) for a review. During this period, the population may not have been able to inhabit the JPN area (Figure 9a). After the last glacial period, temperatures gradually increased and peaked ~6,000 years ago, 2–3°C higher than at present in Japan (Schöne et al., 2004). SST warming enabled the *P. fucata* population to extend northward and to reach the Japanese mainland during this period (Figure 9b). As the SST gradually decreased to its present level, individuals able to tolerate lower water temperatures remained in the JPN area and formed the northern population (Figure 9c). Environmental gradients, especially SST, caused a genetic discontinuity between the modern northern and southern populations. Meanwhile, artificial fertilization experiments indicate that speciation has not occurred between the populations (Atsumi et al., 2004).

Population patterns along the Kuroshio Current in the western Pacific vary among organisms. In particular, diversity of coral species diminishes drastically in the Japanese mainland compared to the Nansei Archipelago (Veron & Minchin, 1992). In other words, the Nansei area is the northern limit of distribution for many coral species, irrespective of their pelagic larval duration. Marine shallow‐water species show common population structure, being divided into southern (Nansei Islands) and northern (Japanese mainland) populations (Chang et al., 2017; Ogoh & Ohmiya, 2005; Yamazaki et al., 2017). In this study, we demonstrated the population structure of a marine bivalve species. Despite their long‐dispersal potential, *P. fucata* is genetically differentiated due to an environmental barrier in the western Pacific. Evidence from this study suggests that the present population distribution was shaped by past environmental changes and by the adaptive capability of the animal. This perspective can be generalized to explain varied population patterns of marine organisms that may have different thermal tolerances and evolutionary consequences. Fossil records indicate that 6,000 years ago, tropical fauna, including hermatypic corals and mollusk species were distributed at higher latitudes (~35°N) (Hoshino, 1967; Matsushima, 1984; Yabe & Sugiyama, 1932) (Figure 9b). Some tropical species retreated to the south as the SST decreased to present levels, while others colonized the JPN area to form northern populations, presumably due to tolerance of lower temperatures.

Our genome‐wide analysis detected genetic introgression of the northern into the southern population (Figure 8). Gene flow, opposite the Kuroshio Current, might be due to artificial introductions. It is known that pearl oysters were brought from the Japanese mainland to Amami Island (NAN_18) and Okinawa Island (NAN_19) for aquaculture purposes in the past. Although pearl farming using *P. fucata* is not continuing at present, hybridization has occurred in these areas and genetic attributes have been retained in the NAN population.

In terms of conservation of *P. fucata*, an important aquaculture species for pearl farming, our results provide essential information about population structure. In order to conserve genetic diversity of the species, artificial admixture for pearl farming should be planned carefully based on natural population structure. In particular, animals from the southern population should not be introduced for pearl farming in the Japanese mainland, or vice versa, to avoid losing their genetic uniqueness. As the northern and southern populations may be adapted different environmental conditions, it is beneficial for sustainable pearl farming to conserve genetic diversity that might be able to overcome climatic changes in the future.

## 5 CONCLUSIONS

Using genome‐wide genotyping data, we determined the population structure of *Pinctada fucata* in the Indo‐Pacific area. Population isolation by geographic barriers is evident in the Indian Ocean (MMR) and a local lagoon in Kamikoshiki Island (JPN_17), whereas environmental gradients can shape large‐scale population differentiation in the western Pacific (northern and southern populations). Genomic loci that may be associated with adaptation to the local environment were identified. The present study highlights demographic history affected by environmental changes that explain the extant marine population in the western Pacific. In addition, the population structure demonstrated by the present study should provide essential information for genetic conservation of the pearl oyster.

## CONFLICT OF INTEREST

None declared.

## Data Availability

SNP dataset is available on DRYAD (https://doi.org/10.5061/dryad.qnk98sfbv) (Takeuchi, 2019).
