# Supplementary material for: Divergent northern and southern populations and demographic history of the pearl oyster in the western Pacific revealed with genomic SNPs
Source: Evol Appl. 2020 Jan 8;13(4):837–53. doi: 10.1111/eva.12905 (PMC7086055; doi:10.1111/eva.12905)
Supplement: Supplementary file 1 [file EVA-13-837-s001.pdf]

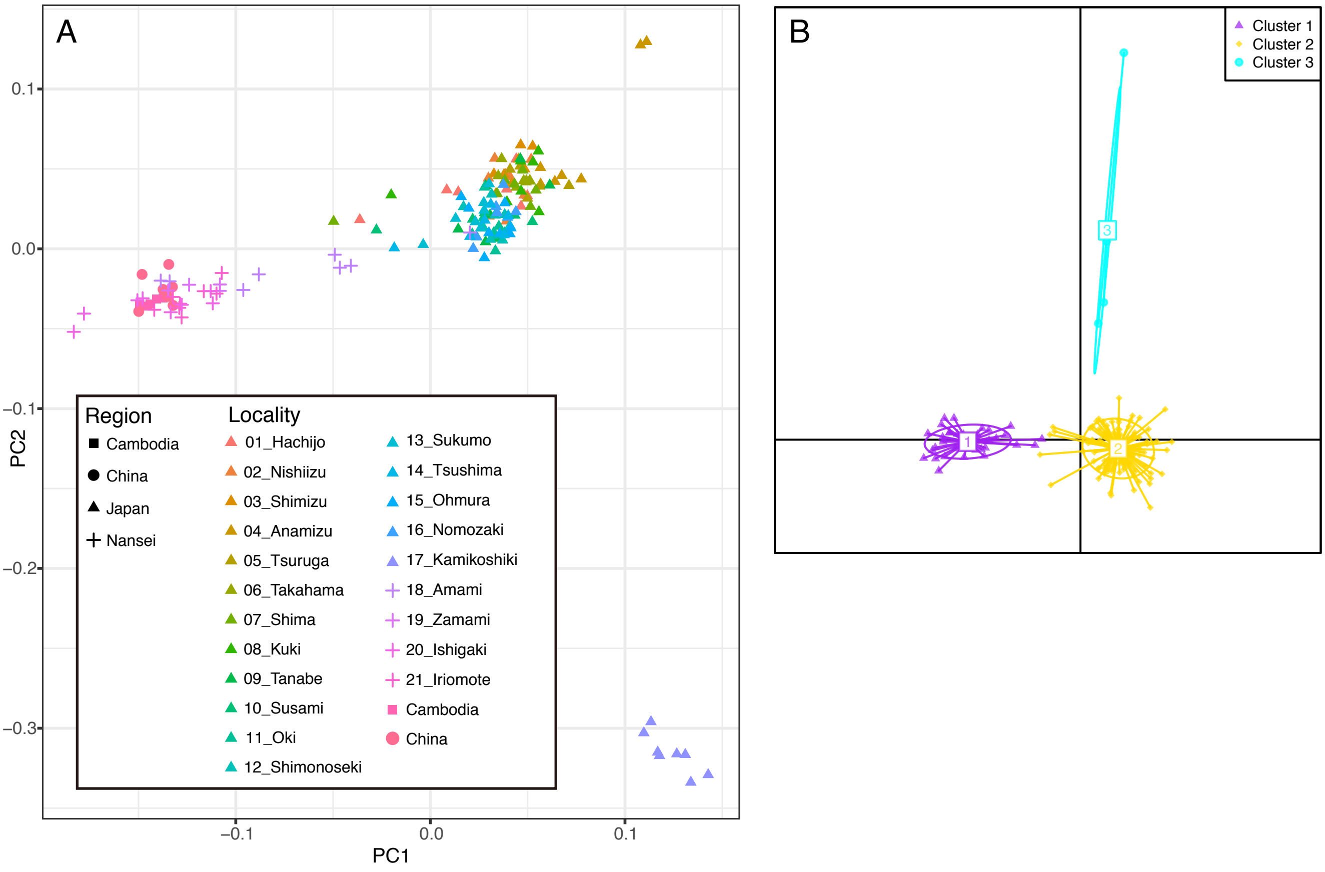

Supplementary Figure 1. Southern and northern populations of *P. fucata* in the western Pacific identified by PCA and DAPC. A) PCA of the western Pacific samples. B) DAPC of the western Pacific samples. Cluster 1 and 2 represent northern and southern populations, respectively. Cluster 3 includes individuals from Kamikoshiki Island (JPN_17).

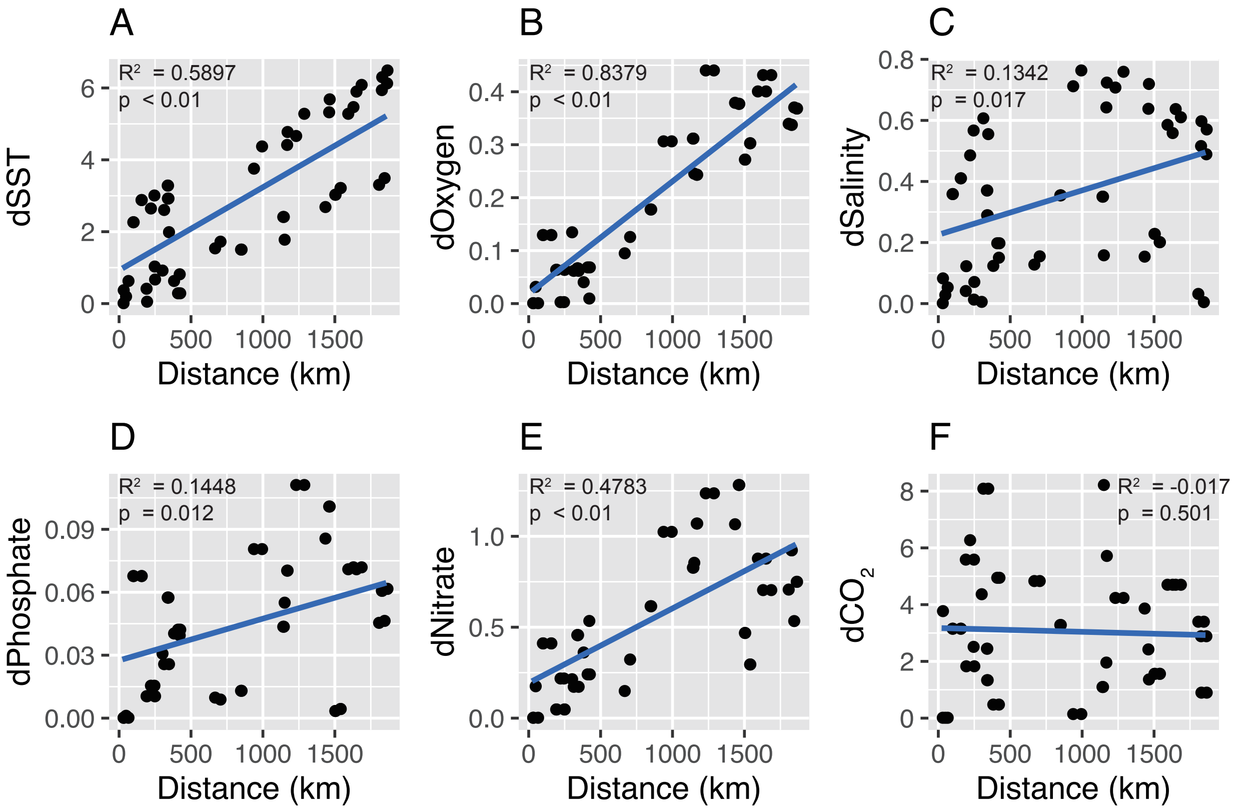

Supplementary Figure 2. Correlation between distance and difference in environmental factors among localities along the Kuroshio Current. Regression lines are shown in blue. Adjusted R-squared and p-values for Mantel tests are also indicated.

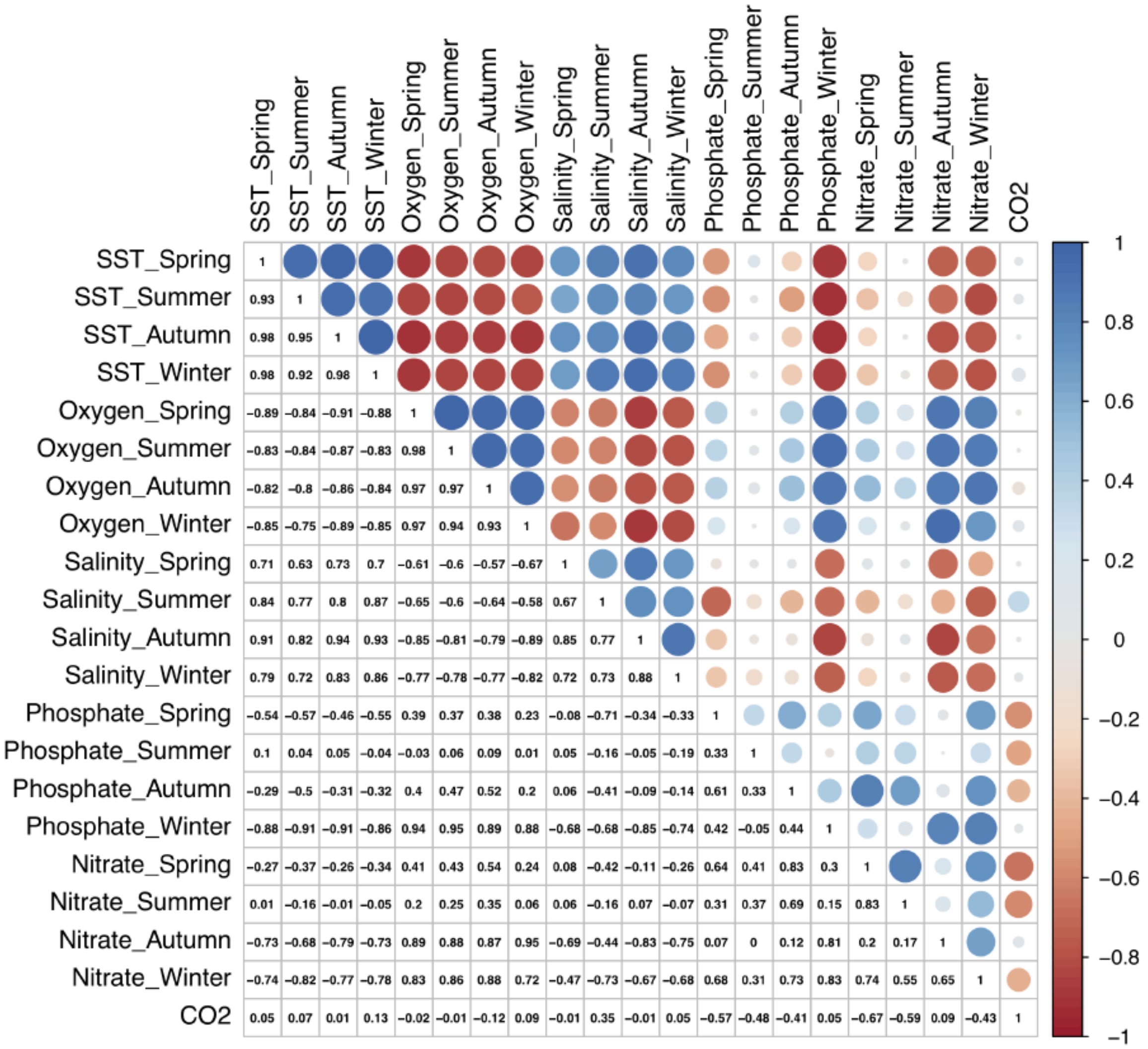

Supplementary Figure 3. Pairwise correlation among environmental factors. Pearson's correlation coefficients are shown in the lower left. Colored circles in the upper right visualize the correlation coefficients.

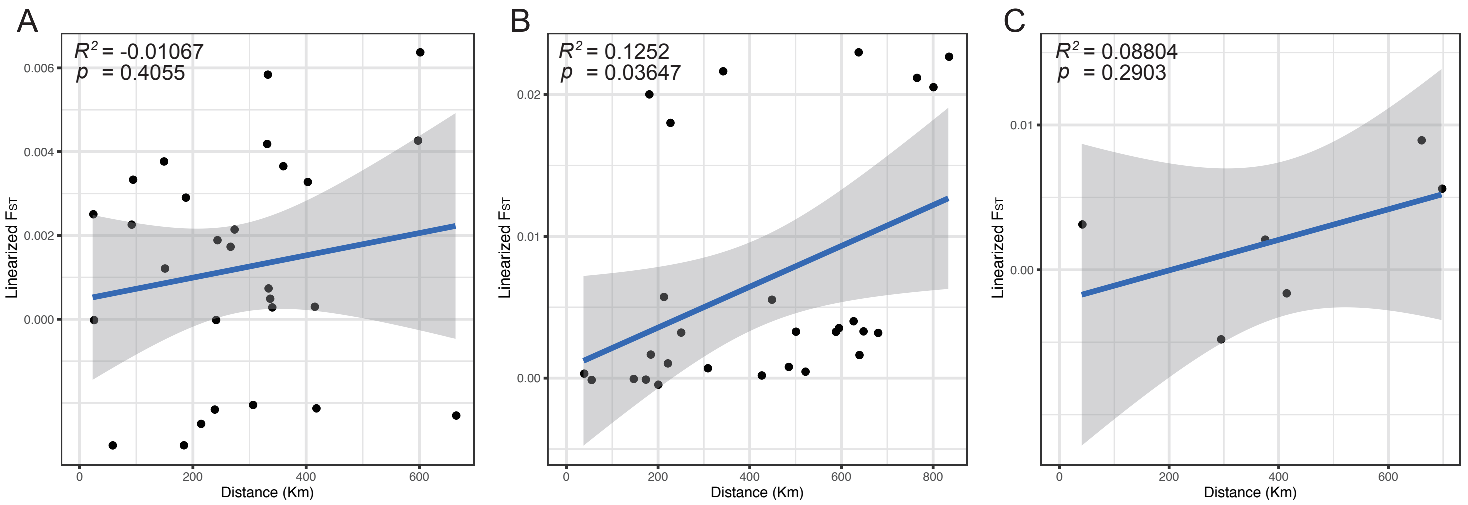

Supplementary Figure 4. Assessment of Isolation-by-distance (IBD). A) Pacific Japan, B) Japan Sea, and C) Nansei regions. IBD is less evident in these regions. Dots indicate pairwise comparisons. Regression lines are shown in blue and 95% confidence limits are shown in gray. P-values are calculated using simple Mantel test.
