# Supplementary material for: Divergent northern and southern populations and demographic history of the pearl oyster in the western Pacific revealed with genomic SNPs
Source: Evol Appl. 2020 Jan 8;13(4):837–53. doi: 10.1111/eva.12905 (PMC7086055; doi:10.1111/eva.12905)
Supplement: Supplementary file 4 [file EVA-13-837-s004.pdf]

Supplementary Note

Effect of Isolation-by-distance (IBD)

Prior to testing IBD, we checked the correlations between distance and gradients of environmental factors. First, we retrieved environmental and distance data from localities along the Kuroshio Current (JPN_1, 2, 3, 7, 8, 9, 10, 13, NAN_18, 19, 20, and 21), because direct distances between Japanese localities along the Pacific coast and in the Japan Sea coast do not represent the distances that marine organisms must migrate. Then, we tested the correlation between distance and differences in environmental factors (annual means) among localities. Except for CO_2, distance and environmental factors are significantly correlated ($p \leq 0.05$) (Supplementary Figure 2). This is mainly because the sampling points span a broad latitudinal range, causing significant gradients in environmental variables such as sea surface temperature (SST) and oxygen. Therefore, IBD cannot be evaluated independently from environmental factors, using data along the Kuroshio Current.

In order to minimize the influence of environmental gradients, we prepared three regional datasets: Japan Pacific (JPN_1, 2, 3, 7, 8, 9, 10, and 13), Japan Sea (JPN_4, 5, 6, 11, 12, 14, 15, 16, 17), and Nansei (NAN_18-21) regions. By analyzing each of these, the effect of the Japanese mainland as a geographic barrier that separates the Pacific Ocean and the Japan Sea was also reduced.

An ecological distance matrix for each environmental factor was generated by calculating pairwise differences between sampling localities. Simple Mantel tests for linearized F_{ST} ($F_{ST}/(1 - F_{ST})$) and ecological factors were assessed in the R ecodist

package (Supplementary Figure 4). Correlations were not significant in the Japanese Pacific (p=0.40) and the Nansei (p=0.29) regions, while they were marginally significant in the Japan Sea (p=0.03). The correlation in the Japan Sea data, however, is mainly due to genetic differentiation found in two populations at Anamizu (JPN_4) and Kamikoshiki Island (JPN_17), where geographic barriers are much more significant than IBD. Overall, IBD does not seem to be an important factor for genetic differentiation of *P. fucata*, at least in the regions examined.
